# Supplementary material for: Discovery and application of insertion-deletion (INDEL) polymorphisms for QTL mapping of early life-history traits in Atlantic salmon
Source: BMC Genomics. 2010 Mar 8;11:156. doi: 10.1186/1471-2164-11-156 (PMC2838853; doi:10.1186/1471-2164-11-156)
Supplement: Additional file 2 — Information on developed 76 locus single-run INDEL panel in Atlantic salmon. Information on fluorescence labeling, primer concentrations, PCR pooling and links to alignments, INDEL motifs and GENESCAN (Burge and Karlin 1997) predictions of genes/exons are available in html format. [file 1471-2164-11-156-S2.ZIP › Additionalfile2/snpsummary21188.html]

```
Cluster 11664 Contig 1

prev  Summary    Contig List  next
```

Size of Consensus sequence = 928

Number of sequences = 4

Minimum redundancy = 1

Key

A gi|85154880|gb|DW588579.1|DW588579 sbra1-005AA11.g1\_#0021.0 DIAS\_SBRA Salmo salar cDNA 5', mRNA sequence  
B gi|117297733|gb|EG647546.1|EG647546 SGP258721 Atlantic salmon normalized Brain cDNA library Salmo salar cDNA clone NB1-0826 5', mRNA sequence  
C gi|117298143|gb|EG647956.1|EG647956 SGP259545 Atlantic salmon normalized Brain cDNA library Salmo salar cDNA clone NB1-1282 5', mRNA sequence  
D gi|83640262|gb|DW006852.1|DW006852 SGP296350 Atlantic salmon Brain cDNA library Salmo salar cDNA clone HJ4-3551 5', mRNA sequence

5 SNPs detected

A B C D  cosegregation weighted

29 T G T .   3/5 45.00
159 T - T .   3/5 45.00
160 G - G .   3/5 45.00
323 G G T .   1/5 15.00
461 . C C T   1/5 15.00
